# Social inequalities in patient experiences with general practice and in access to specialists: the population-based HUNT Study

**DOI:** 10.1186/1472-6963-13-240

**Published:** 2013-07-01

**Authors:** Eirik Vikum, Roar Johnsen, Steinar Krokstad

**Affiliations:** 1 Department of Public Health and General Practice, Faculty of Medicine, Norwegian University of Science and Technology (NTNU), Trondheim, Norway; 2 HUNT Research Centre, Department of Public Health and General Practice, Faculty of Medicine, Norwegian University of Science and Technology (NTNU), Trondheim, Norway; 3 Levanger Hospital, Nord-Trøndelag Health Authority, Levanger, Norway

**Keywords:** Social inequalities, Socioeconomic, Health services research, Health care utilization, General practice, Patient experiences, Norway

## Abstract

**Background:**

In countries with gatekeeping and equitable access to general practitioners (GPs), social inequalities in GP-patient interaction could be an important mechanism by which inequalities in access to medical specialists arise. The aim of this study was to investigate whether socioeconomic inequalities in experiences with general practice are associated with socioeconomic inequalities in access to specialist services.

**Methods:**

The study included 6,067 participants in the third survey of the Nord-Trøndelag Health Study (HUNT3, 2006–08) who were asked to evaluate their experiences with primary care and their regular general practitioner in Norway. Self-reported data on health status and number of visits to GP and specialist services in the last 12 months were included in the study. Socioeconomic status was measured by education and household income and rescaled to relative index of inequality (RII). Relative risks were calculated using Poisson regression.

**Results:**

We found that a majority of patients reported positive experiences with general practice. Low socioeconomic status (SES) and male gender were associated with negative experiences. Patient experiences both directly and indirectly related to referrals were associated with the probability and quantity of specialist utilization: perception of low subjective influence on decisions about choice of medical care was associated with *lower* probability and quantity of specialist utilization, whereas desire to change the regular GP or to use GPs other than the regular GP and critical evaluations of the GP were associated with *higher* specialist consultation frequency. However, the level of education-related inequity in access to specialists was not sensitive to adjustment by survey responses.

**Conclusion:**

Patient experiences with general practice were associated with the patients’ level of utilization of specialist services. There are socioeconomic inequalities in patient experiences with general practice, however the aspects measured in this study do not explain the observed socioeconomic inequity in access to specialists.

## Background

In many Western countries there have been consistent reports of socioeconomic equity in the utilization of general practitioner (GP) care and inequity in the utilization of specialist services [1,2]. The same pattern has been found in several countries, including Norway [3-5], where GPs are gatekeepers to specialist services and therefore exert large influence on specialist utilization.

The decision of GPs to refer patients to specialist services is likely to be a central node in the mechanisms leading to inequity in specialist utilization. Socioeconomic inequalities in referrals from general practitioners to specialist care have been found in studies in Great Britain and Denmark [6,7]. One study showed that Australian patients with high socioeconomic status (SES) were more likely to be assigned to diagnostic tests by general practitioners [8].

Little is known about the mechanisms by which socioeconomic inequalities in referrals to specialist services arise. Studies have shown that a significant share of referrals from GPs to specialists may be medically inappropriate [9]. Little et al. 2004 found that GPs decisions to refer are influenced by patient expectations and by perceived patient pressure, and that a large minority of referrals were by the doctors thought of as founded on little or no need [10]. That indicates flexibility on the part of general practitioners to accommodate patient demands, which gives room for inequalities in referrals to arise. Previous literature has identified three mechanisms for the influence of socioeconomic status on the outcomes of general practitioner consultations, excluding health status: (1) the social distance between doctor and patient, influencing communication, (2) status-dependent differences in health knowledge and beliefs influencing health related behavior among patients, and (3) the professional power of the doctor [8,11]. These mechanisms may influence patient satisfaction with GP consultations. However we know of no studies that have investigated socioeconomic inequalities in patient experiences with GP-patient interaction in a strict gatekeeping system and their association with referrals to specialist services.

In the Norwegian context socioeconomic inequity in specialist utilization can be assumed to be a good approximation of socioeconomic inequalities in referrals, as GP utilization levels are high regardless of socioeconomic status [3,4] and GPs are gatekeepers to specialist services. While socioeconomic discrimination in waiting times to secondary care has been reported, [12] fees for specialist services in Norway are fixed at generally low levels with a cap on yearly health care spending per person, such that barriers to specialist utilization can be considered few once a patient has been referred.

### Setting: general practice in Norway

Nearly all Norwegian citizens are assigned to a regular GP within the list-based system that was introduced in 2001. Most general practitioners are self-employed on contracts with municipalities. A majority of medical specialist practices outside hospitals are private and operate on contracts with the public Regional Health Authorities. General practitioners are gatekeepers to all specialist care and elective hospital treatment that is reimbursed by the National Insurance Scheme. Co-payments for publicly reimbursed specialist and general practitioner care are fixed, and low.

### Aim

The aim of this study was to describe socioeconomic inequalities in patient experiences with general practice and to investigate whether socioeconomic inequalities in experiences with general practice explain socioeconomic inequalities in access to specialist services.

## Methods

### Data sources

The Nord-Trøndelag Health Study (HUNT) is a total county population based health study, and maintains a unique database of medical histories collected during three cross-sectional surveys: HUNT1 (1984–86), HUNT2 (1995–97) and HUNT3 (2006–08) [13]. Applications for access to the data are considered by the HUNT Research Centre in Levanger, Norway.

Nord-Trøndelag is one of 19 counties in Norway, situated in the middle of the country with a stable and homogenous population of approximately 130,000 inhabitants. The county lacks large cities.

**Figure 1:**
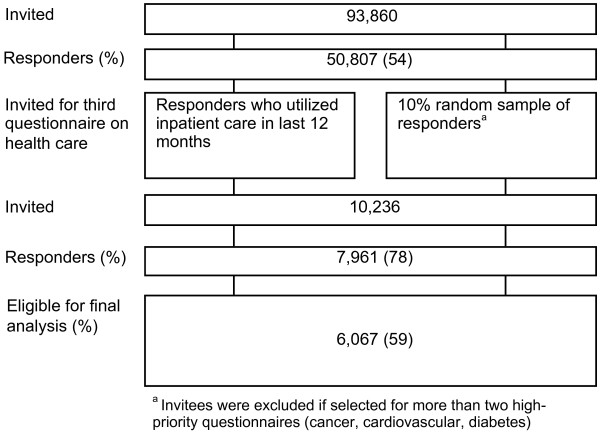
**Schematic overview of sample composition.** The Nord-Trøndelag Health Study, HUNT3 2006–08.

All persons aged 20 years and above in the county of Nord-Trøndelag were invited to participate in the HUNT 3 Survey (2006–08). Out of 93,860 persons invited, 54% (50,807) responded to the first and second questionnaires of the survey. A new selection of responders was made for the third questionnaire concerning health services, including a 10% random sample of all responders, and all responders who had been hospitalised in the last year. Of 10,236 persons selected, 7,961 (78%) responded. 1,894 persons were subsequently excluded due to incomplete data from the HUNT3 questionnaires. See Figure 1 for a schematic overview of the sample selection. There were no incentives of any kind to survey participants. For further details on data collection, see the cohort profile for the HUNT surveys [13] and the nonparticipation study for HUNT3 [14].

Data on household income and level of education was appended from national register data from Statistics Norway (SSB) using the unique personal identity number given all Norwegian citizens. We restricted analyses to men and women 20 years and older.

### Variables

Responders were asked a range of questions concerning evaluations of their general practitioner, utilization patterns and perception of the possibility of referral to specialist services. A selection of ten questions judged to be most relevant to the study was made. The full questionnaire is available at http://www.ntnu.edu/hunt/data/que. The responses to the questions were then dichotomized, whereby the responses presented in this study were coded as 1, and the respective reciprocal responses were coded as 0.

Two indicators of health care utilization were employed. Respondents were asked how many times in the past 12 months they had visited a general practitioner and medical specialist. Where count utilization data were missing from the third questionnaire, dichotomous utilization data from the first questionnaire was used where available. Self-reported health was measured by four response alternatives: “very good”, “good”, “not so good” and “poor”. Other health indicators were available, but were not used in order to preserve sample size.

Educational level, obtained from Statistics Norway and following the Norwegian Standard Classification of Education (NUS), was coded into three levels of highest educational level attained: primary (primary and secondary school), secondary (high school or equivalent) and tertiary (college and/or university), primary used as reference category where relevant.

Disposable income per equivalent adult was calculated using household income after tax from 2007 based on tax registry data from Statistics Norway. Where available, spouses and cohabiting persons over 18 years were given a weight of 0.5, and children up to 18 years a weight of 0.3. The lowest income quartile was used as the reference.

Municipality of residence for each respondent was included in the analyses as a measure to control for regional differences in access to health services. The variable subdivided respondents into three categories: municipalities with less than 10,000 inhabitants (*n = 19*), large (more than 10,000 inhabitants) municipalities without hospital (*n = 3*), and large municipalities with hospital (*n = 2*).

### Relative index of inequality

The relative index of inequality (RII) is a summary measure of relative inequality in an outcome along a socioeconomic scale, and takes into account both the relative sizes of the subgroups in a study population and their relative socioeconomic position [15]. The index was calculated, in the case of education, by creating a new variable that ordered the educational groups from highest to lowest level of education. Ranging between 0 and 1, each group was given a score based on the midpoint of its cumulative percentage share of the population. 25% of participants had tertiary education and were in this variable coded 0.125 (0.25/2), 54% had secondary education and were coded 0.52 (0.25+(0.54/2)), and finally the 21% with primary education were coded 0.895 (1-(0.21/2)). The income variable was structured differently, and in order to create a corresponding measure the RII score variable was calculated from household income quartiles comprising 25% of the study population.

### Statistical analysis

Estimates were calculated using Poisson regression with robust error variance for dichotomous outcomes [16] and regular Poisson regression for counts. Risk ratios were estimated for dichotomous outcomes due to the large variation in average utilization rates (see Table 1), which would make the odds ratios for different utilization indicators difficult to compare. Predicted probabilities (PP) were calculated at the means of the other variables. Confidence intervals were calculated at the 95% level. Health care utilization was needs-adjusted by controlling for age, gender and self-reported health, following common practice [17]. All estimates were adjusted for hospitalization in the past year, in order to control for potential bias from the selection.

**Table 1:** Comparisons of sample features to the HUNT3 population

	**Eligible for this study**		**HUNT3 overall**	
	**Men**	**Women**	**Men**	**Women**
	**% (N)**	**% (N)**	**N (%)**	**N (%)**
	**42 (2,584)**	**58 (3,545)**	**46 (21,500)**	**54 (25,360)**
*Age*				
20-39 years	13	28	20	24
40-59 years	41	38	44	42
≥ 60 years	46	34	36	34
*Education*				
Primary	20	22	20	24
Secondary	60	49	58	48
Tertiary	19	29	22	29
*Self-rated health status*				
Poor	3	2	1	1
Not so good	32	35	22	27
Good	54	50	61	56
Very good	11	13	16	16
*Health care utilization*				
General practitioner	89	92	75	84
Specialist	51	51	34	38
Hospital inpatient care	58	59	11	12

The HUNT Study, 2006–08.

The RII can be interpreted as the probability of an outcome for a hypothetical person with the lowest socioeconomic status compared to the same probability for a hypothetical person with the highest status.

In order to test whether the survey responses represent pathways of association between socioeconomic status and specialist services utilization, educational inequalities in specialist utilization was measured prior to and after adjusting for each of the various dichotomized survey responses, one at a time. If the level of inequality, as measured by RII, remained unchanged after adjusting for a given survey response, the survey response was judged not to be associated with an inequity-generating mechanism.

All analyses were done using Stata IC 12.1.

## Results

Table 1 shows an overview of the educational level, age distribution, self-reported health status and health care utilization in the sample. Due to the survey design, 58% of the sample is comprised of persons that have received inpatient care in the last year. Thus the average age in the sample is relatively high, the self-reported health status relatively poor and health care utilization levels high, compared to average levels for the population of HUNT3 survey responders as a whole.

Table 2 presents an overview of the socioeconomic distribution of health care utilization in the sample, adjusted for age, gender, self-reported health and municipality size. General practitioner utilization levels were very high (90-91%), and the probability of utilization was equitable by household income and education (education RII = 1.02, 95% CI 0.99-1.05). Low education was associated with a higher number of visits to GPs (education RII = 1.06, 95% CI 1.00-1.12). Specialist utilization levels were high (51%) in the sample. The probability of specialist services utilization was distributed in disfavor of persons with lower household income and lower education (education RII = 0.83, 95% CI 0.75-0.91), and lower education and household income were associated with a lower number of visits to specialists (education RII = 0.62, 95% CI 0.57-0.62).

**Table 2:** Utilization of general practitioner and specialist services by education and household income

	**General practitioner**				**Specialist services**			
	**Probability of at least one visit**		**Number of visits**		**Probability of at least one visit**		**Number of visits**	
	**RII**^**a**^	**CI 95%**	**RII**^**a**^	**CI 95%**	**RII**^**a**^	**CI 95%**	**RII**^**a**^	**CI 95%**
Education	1.02	(0.99-1.05)	1.06	(1.00-1.12)	0.83	(0.75-0.91)	0.62	(0.57-0.62)
Household income	1.00	(0.97-1.02)	0.99	(0.94-1.04)	0.87	(0.80-0.96)	0.74	(0.68-0.74)
	PP^a^	CI 95%	PP^a^	CI 95%	PP^a^	CI 95%	PP^a^	CI 95%
Education								
*Primary*	0.91	(0.90-0.92)	3.37	(3.27-3.46)	0.49	(0.47-0.52)	1.26	(1.20-1.26)
*Secondary*	0.91	(0.90-0.92)	3.51	(3.44-3.57)	0.49	(0.48-0.51)	1.42	(1.38-1.42)
*Tertiary*	0.90	(0.88-0.91)	3.21	(3.11-3.31)	0.57	(0.54-0.60)	1.80	(1.73-1.80)
Household income								
*Poorest*	0.91	(0.89-0.92)	3.33	(3.23-3.42)	0.47	(0.45-0.50)	1.25	(1.19-1.25)
*2nd quartile*	0.90	(0.89-0.92)	3.49	(3.40-3.58)	0.51	(0.49-0.54)	1.44	(1.33-1.44)
*3rd quartile*	0.91	(0.89-0.92)	3.45	(3.35-3.54)	0.53	(0.51-0.56)	1.59	(1.52-1.59)
*Richest*	0.91	(0.89-0.92)	3.36	(3.27-3.46)	0.52	(0.50-0.55)	1.58	(1.51-1.58)

^a^ Poisson regression was used for all measures, and with robust error variance for probabilities of at least one visit. Predicted probabilities (PP) were calculated from the corresponding regressions, using categorical variables for education and household income instead of the RII-variable. All estimates were adjusted for self-reported health, age, gender and municipality size.

Relative index of inequality (RII) and predicted probabilities (PP). Men and women aged 20 years and above. N = 6,067. The HUNT Study, 2006–08.

### Socioeconomic inequalities in patient experiences with general practice

Responses to the survey questions included in the study, and the percentage of the sample that gave the given response are presented in Table 3. Measures of association between the given responses and education, household income and male gender are reported as relative index of inequality (education and household income) and relative risk (gender). Overall, responders reported positive experiences with general practice. However, a significant minority reported negative experiences, and all responses were associated with either socioeconomic status or gender, or both.

**Table 3:** Probabilities of survey responses by education, household income and gender

			**Education**		**Household income**		**Gender (male)**		
**Response to survey question**		**%**	**RII**^**b**^	**CI 95%**	**RII**^**b**^	**CI 95%**	**RR**^**b**^	**CI 95%**	**N**
1	My experience with the regular GP has been poor (0–5 / 10 on VAS scale)	12	1.48	(1.13-1.93)	1.07	(0.82-1.40)	1.23	(1.07-1.41)	6067
2	My regular GP has poor understanding of my problems	6	1.34	(0.89-2.02)	1.44	(0.95-2.19)	1.42	(1.15-1.76)	6067
3	My regular GP does not let me participate in decisions about treatment or choice of medical care	11	1.60	(1.21-2.10)	1.15	(0.86-1.53)	1.51	(1.31-1.75)	6067
4	My regular GP does not take me seriously	4	1.58	(0.96-2.59)	1.38	(0.83-2.28)	1.29	(1.00-1.65)	6067
5	My usual doctor is not my regular GP	5	1.56	(1.01-2.42)	1.29	(0.86-1.94)	1.51	(1.20-1.90)	6067
6	I have changed or wanted to change my regular GP	19	0.61	(0.50-0.75)	0.88	(0.72-1.07)	0.75	(0.67-0.84)	6067
7	I have had problems understanding my GP due to language problems	11	3.25	(2.49-4.26)	1.69	(1.28-2.22)	1.06	(0.91-1.23)	6067
8	I have not received the help I asked for from my regular GP in last 12 months	7	1.66	(1.13-2.45)	1.06	(0.70-1.60)	1.13	(0.92-1.40)	4924^a^
9	It has been difficult to get a referral to a specialist	16	2.02	(1.54-2.64)	1.34	(1.00-1.78)	1.17	(1.01-1.35)	4165^a^

^a^ These analyses included only those who reported that this question was relevant to them.

^b^ Relative index of inequality and relative risk calculated using Poisson regression with robust error variance. All estimates were adjusted for age, sex, municipality size and hospitalization within past year.

Men and women aged 20 years and above. The HUNT Study, 2006–08.

Men were more likely to report negative experiences with general practice, but less likely to have changed or wanting to change their regular GP (relative risk (RR) = 0.75, 95% CI 0.67-0.84). Change of regular GP was also associated with having higher education (RII = 0.61, 95% CI 0.50-0.75). 12% reported having had poor experience with their regular GP, and the response was associated with low education (RII = 1.48, 95% CI 1.13-1.93) and being male (RR = 1.23, 95% CI 1.07-1.41).

Eleven percent reported that their regular GP did not let them participate in decisions about treatment or choice of medical care, and the response was associated with being male (RR = 1.51, 95% CI 1.31-1.75) and having low education (RII = 1.58, 95% CI 1.21-2.10). 16% of a subsample reported that it had been difficult to get a referral to a specialist, and the response was associated with lower education (RII 2.02, 95% CI 1.54-2.64), lower household income (RR = 1.34, 95% CI 1.00-1.78), and being male (RR = 1.17, 95% CI 1.01-1.35).

### Associations of patient experiences with specialist utilization

Table 4 shows the association between the given responses and the probability and quantity of specialist utilization. Persons reporting problems understanding their GP were more likely to have seen a specialist (RR = 1.10, 95% CI 1.03-1.18), and likely to have a higher number of consultations with a specialist (RR = 1.10, 95% CI 1.03-1.17). Having changed or wanting to change the regular GP was associated with a higher number of specialist consultations (RR = 1.15, 95% CI 1.09-1.21). Reporting that it is difficult to get a referral was associated with a lower probability of access to specialists (RR = 0.91, 95% CI 0.85-0.98) and a lower number of consultations (RR = 0.83, 95% CI 0.78-0.89). Feeling left out of decisions concerning treatment or choice of medical care was also associated with a lower number of specialist consultations (RR = 0.88, 95% CI 0.82-0.94).

**Table 4:** Associations between survey responses and utilization of specialists

			**Probability of specialist visit**		**Number of visits to specialist**		
		**%**	**RR**^**b**^	**CI 95%**	**RR**^**b**^	**CI 95%**	
1	My experience with the regular GP has been poor (0–5 / 10 on VAS scale)	12	0.97	(0.90-1.04)	1.00	(0.93-1.06)	6067
2	My regular GP has poor understanding of my problems	6	1.01	(0.92-1.11)	1.13	(1.04-1.23)	6067
3	My regular GP does not let me participate in decisions about treatment or choice of medical care	11	0.95	(0.88-1.03)	0.88	(0.82-0.94)	6067
4	My regular GP does not take me seriously	4	1.04	(0.93-1.16)	1.12	(1.02-1.24)	6067
5	My usual doctor is not my regular GP	5	1.08	(0.97-1.20)	1.36	(1.24-1.49)	6067
6	I changed or wanted to change my regular GP	19	1.04	(0.98-1.10)	1.15	(1.09-1.21)	6067
7	I have had problems understanding my GP due to language problems	11	1.10	(1.03-1.18)	1.10	(1.03-1.17)	6067
8	I have not received the help I asked for from my regular GP in last 12 months	7	1.03	(0.94-1.12)	1.06	(0.98-1.15)	4924^a^
9	It has been difficult to get a referral to a specialist	16	0.91	(0.85-0.98)	0.83	(0.78-0.89)	4165^a^

^a^ These analyses included only those who reported that this question was relevant to them.

^b^ Relative risks calculated using Poisson regression, with robust error variance for probability of specialist visit. All estimates were adjusted for age, sex, self-reported health, municipality size and hospitalization within past year.

Men and women aged 20 years and above. The HUNT Study, 2006–08.

### Associations of socioeconomic inequalities in patient experiences with inequalities in specialist utilization

In Table 5 education-related inequalities in the number of specialist visits in the sample, before and after adjusting for the each of the survey questions individually are shown. Adjusting for the responses individually did not change the magnitude of the education-related RII, and only to a very minor degree for reported language problems (unadjusted RII = 0.62, adjusted RII = 0.61) and reported difficulty getting a referral to a specialist (unadjusted RII = 0.66, adjusted RII = 0.67).

**Table 5:** Educational inequity in the predicted number of specialist visits before and after adjusting for survey responses

			**Not adjusting for the given survey response**		**Adjusting for the given survey response**		
		**%**	**RII**^**b**^	**CI 95%**	**RII**^**b**^	**CI 95%**	
1	My experience with the regular GP has been poor (0–5 / 10 on VAS scale)	12	0.62	(0.57-0.67)	0.62	(0.57-0.67)	6067
2	My regular GP has poor understanding of my problems	6	0.62	(0.57-0.67)	0.62	(0.57-0.68)	6067
3	My regular GP does not let me participate in decisions about treatment or choice of medical care	11	0.62	(0.57-0.67)	0.62	(0.57-0.68)	6067
4	My regular GP does not take me seriously	4	0.62	(0.57-0.67)	0.62	(0.57-0.67)	6067
5	My usual doctor is not my regular GP	5	0.62	(0.57-0.67)	0.62	(0.57-0.67)	6067
6	I changed or wanted to change my regular GP	19	0.62	(0.57-0.67)	0.63	(0.58-0.68)	6067
7	I have had problems understanding my GP due to language problems	11	0.62	(0.57-0.67)	0.61	(0.56-0.66)	6067
8	I have not received the help I asked for from my regular GP in last 12 months	7	0.64	(0.59-0.70)	0.64	(0.59-0.70)	4924^a^
9	It has been difficult to get a referral to a specialist	16	0.66	(0.61-0.72)	0.67	(0.61-0.73)	4165^a^

^a^ These analyses included only those who reported that this question was relevant to them.

^b^ Education-related relative index of inequality calculated using Poisson regression with robust error variance. All estimates were adjusted for age, self-reported health, sex, municipality size and hospitalization within past year.

Men and women aged 20 years and above. The HUNT Study, 2006–08.

### Missing

Non-response to the third questionnaire and exclusion in this study due to missing values was associated with low education, being male, and ages 20–39 and ≥70 years.

## Discussion

A majority of patients reported positive experiences with general practice and stability in the GP-patient relationship. A significant minority reported negative experiences, however, and persons with low education, low household income and of male gender were more likely to be in that group. In spite of higher satisfaction on average, women and persons with high education were more likely to have changed or wanting to change their regular GP.

Furthermore, we found that patient experiences both directly and indirectly related to referrals were associated with the probability and quantity of specialist utilization. There were three general groups of associations: (1) perception of low influence on decisions about choice of medical care was associated with lower probability and quantity of specialist utilization, and (2) desire to change the regular GP or to use GPs other than the regular one was associated with higher specialist consultation frequency, as were (3) evaluations that were critical of the GP.

In spite of the presence of both socioeconomic inequalities in experiences with general practice and educational inequity in specialist utilization in the sample, however, none of the survey responses were found to be important mediators of the association between education and the probability of access to specialist services.

### Strengths and limitations

The strengths of this study include the relatively large sample size and the population-based data. The education and income data from Statistics Norway are considered accurate.

The HUNT3 Survey of 2006–08 had a response level of 54%, and nonparticipation was associated with socioeconomic status, morbidity, lifestyle factors and to some extent health care utilization [14]. The nonparticipation pattern could contribute to an underestimation of socioeconomic inequalities in specialist services utilization, which introduces some uncertainty concerning the validity of our findings. Due to the original selection, non-response and missing variables, the present study population differs from the original HUNT3 sample and the general population in the county of Nord-Trøndelag, particularly in regard to morbidity levels, utilization levels and age composition. However, the general pattern of inequality found in this study, showing socioeconomic equity in general practitioner utilization and socioeconomic inequity in specialist utilization is the same as reported in recent Norwegian [3,5,18] and international studies [1,2], including a study using HUNT3 data [4]. Thus the sample of responders is relevant in uncovering mechanisms generating inequity in access to specialist care.

We use the concepts equity and inequity to describe income- and education-related inequalities in health care utilization that are adjusted for the need for health care [17]. However, the needs-adjustment in this study by age and self-reported health is only an approximation of objective need. Reliance on self-reported health for needs-adjustment could lead to underestimation of pro-educated inequity in health care utilization [19]. Other health status indicators were available to the analyses, but were not employed in order to preserve sample size. Besides, self-reported health has been found to represent a comprehensive health and illness measure [20], and measures of need determined from disease reporting or identified by clinical examinations do not necessarily correspond with demand for specialist services [21]. A sensitivity analysis showed that the outcome estimates were not sensitive to additional adjustment by a dichotomous measure of long-term functional impairment.

The measures of health care utilization employed in this study pertain to the quantity of health care consultations, and do not allow a description of possible socioeconomic inequalities in treatment intensity or quality.

### Previous literature

A few studies have been conducted that identify potential associations with socioeconomic inequalities in referrals to specialist services. McBride et al. 2010 found that socioeconomic inequalities in referrals were more prevalent for less critical conditions and in the absence explicit guidance [6]. A series of U.S. studies found racial inequalities in perceptions of the potential outcomes of arthroplasty, leading to racial inequalities in patient preferences for specialist treatment [22] and possibly inequalities in rates of surgical treatment. The same mechanisms could in theory be generating income- and education-related inequalities in referrals. Education-related inequalities in health care seeking behavior have been shown [23], potentially leading to social inequalities in the conditions or reasons for which GPs are visited in the first place. Another branch of research has shown socioeconomic inequalities in various aspects of doctor-patient communication [24], uncovering potential mechanisms for inequalities in referrals to arise. One such study found that college graduates were more likely to discuss cancer screening with their primary physician [25].

### Interpretation

While low socioeconomic status and male gender were associated with the reporting of negative experiences with general practice, large parts of all social groups reported positive experiences with their GP. Thus this study does not describe socioeconomic inequalities that dominate the way general practice is experienced or used in the various social groups, but inequalities that concern a significant minority within each group.

Given the socioeconomic inequalities in patient experiences with general practice, the associations between the same patient experiences and specialist utilization found in this study represent possible pathways of causation between socioeconomic status and the utilization of specialist services. However, we found that the socioeconomic distribution of the patient experiences that were measured was to a negligible degree associated with the level of education-related inequity in access to specialists. Education-related inequity in specialist access was found also in subsamples where all responders reported that they are able to partake in decisions about choice of care, as well as among those who report that getting a referral is not difficult. Thus to the extent that there are mechanisms in general practice that generate inequity in specialist utilization, they are not mechanisms that generate widespread discontent with GP-patient interaction as measured by the variables in this study.

Our results have implications for further research on the determinants of inequity in specialist utilization. If the mechanisms generating socioeconomic inequalities in referrals are not associated with discontent with the GP-patient relationship, as our study might suggest, socioeconomic discrimination in the GP-patient relationship has to be subtle and not run contrary to patient expectations of being referred in low-SES groups. Inequity-generating mechanisms occurring before the GP-patient interaction would be compatible with our findings. For example, socioeconomic inequalities in health care seeking behavior have been shown [23]. If low-SES patients to a greater extent seek GP consultations for issues that are easily resolved in general practice or that are not judged to warrant specialist-level investigations by the patient, that could explain higher rates of referrals among high-SES patients without an association with discontent in low-SES patients. Socioeconomic disparities in patient preferences for specialist services would also be compatible with our findings.

## Conclusion

Patient experiences with general practice are associated with the patients’ level of utilization of specialist services. There are socioeconomic inequalities in patient experiences with general practice, however the aspects measured in this study do not explain the observed socioeconomic inequity in utilization of specialist services.

## Competing interests

The authors declare that they have no competing interests.

## Authors’ contributions

EV proposed the design, performed statistical analyses and wrote the manuscript. SK and RJ initiated and supervised the study, and revised the manuscript. All authors read and approved the final manuscript.

## Pre-publication history

The pre-publication history for this paper can be accessed here:

http://www.biomedcentral.com/1472-6963/13/240/prepub
